# A Novel System for Functional Determination of Variants of Uncertain Significance using Deep Convolutional Neural Networks

**DOI:** 10.1038/s41598-020-61173-1

**Published:** 2020-03-06

**Authors:** Lior Zimmerman, Ori Zelichov, Arie Aizenmann, Zohar Barbash, Michael Vidne, Gabi Tarcic

**Affiliations:** NovellusDx, Jerusalem, 9112001 Israel

**Keywords:** Cellular signalling networks, Mathematics and computing

## Abstract

Many drugs are developed for commonly occurring, well studied cancer drivers such as vemurafenib for BRAF V600E and erlotinib for EGFR exon 19 mutations. However, most tumors also harbor mutations which have an uncertain role in disease formation, commonly called Variants of Uncertain Significance (VUS), which are not studied or characterized and could play a significant role in drug resistance and relapse. Therefore, the determination of the functional significance of VUS and their response to Molecularly Targeted Agents (MTA) is essential for developing new drugs and predicting response of patients. Here we present a multi-scale deep convolutional neural network (DCNN) architecture combined with an *in-vitro* functional assay to investigate the functional role of VUS and their response to MTA’s. Our method achieved high accuracy and precision on a hold-out set of examples (0.98 mean AUC for all tested genes) and was used to predict the oncogenicity of 195 VUS in 6 genes. 63 (32%) of the assayed VUS’s were classified as pathway activating, many of them to a similar extent as known driver mutations. Finally, we show that responses of various mutations to FDA approved MTAs are accurately predicted by our platform in a dose dependent manner. Taken together this novel system can uncover the treatable mutational landscape of a drug and be a useful tool in drug development.

## Introduction

Precision Medicine, the paradigm proposing that treatment should be tailored to patients according to the individual molecular characteristics of their tumor, is gaining more and more evidence^1^. This paradigm is fueled by the rapid progress in sequencing technologies of tumor samples and the inception of several projects such as the Catalogue of Somatic Mutations In Cancer (COSMIC)^2^ and The Cancer Genome Atlas^3^, all of which aim to identify actionable alterations that could lead to novel therapies, and by the growing number of FDA approved targeted therapies. One of the sparks that ignited the precision medicine paradigm was the identification of the BCR/Abl fusion event as a primary cancer driving mutation in Chronic Myeloid Leukemia^4^ and the subsequent development of imatinib^5^ in 1998 that resulted in dramatic clinical responses and FDA approval in 2001. Since the commercialization of imatinib, dozens of MTAs have been developed for various indications– from kinase inhibitors to monoclonal antibodies^6^. While many malignant genetic alterations have been thoroughly characterized and can be successfully treated^7^, tumor genetic screening frequently discovers rare mutations that have uncertain significance for disease formation and therefore pose a challenge for developing targeted therapies^8^ and establishing reliable eligibility criteria for clinical trials^9^. For example, In the TCGA database, more than 30% of lung adenocarcinoma samples with either nonsense or missense mutations in EGFR have mutations at positions that are not frequently observed^10^. Moreover, a similar analysis for all cancers showed that this proportion increases to 45%. The variants found in those analyses however, are both driver and passenger mutations which may not participate in oncogenesis. Indeed, most drugs are clinically validated only against a handful of mutations (for example, erlotinib for EGFR exon 19 mutations in lung cancer^11^) and occasionally are given off-label in cases where there is some basis of actionability, with limited success^12^.

Given the abundance of VUS in these datasets, a strategy that includes accurate characterization of the activity of VUS and their response to MTAs could provide significant benefit to drug development and increase the success rates of clinical trials. However, this requires methods to tackle the challenging task of deciphering the role of VUS in oncogenesis. Although VUS are frequently abundant in tumors, each individual VUS is rare^13^. Therefore, elucidating the role of each of those mutations from sequencing data alone is not feasible using computational tools due to their rarity and therefore lack of sufficient power for statistical analysis. In many genes, most of the oncogenic mutations are located on functionally important positions which are colloquially termed “hotspots”. Although the occurrence of a mutation in hotspots increases the likelihood of it being oncogenic, it is neither a necessary nor sufficient condition for determining its role in oncogenicity and therefore, serve as a sub-optimal predictor. (One such example is BRAF V600M which is located in a functionally important residue, but was found only to be an intermediate pathway activator compared to V600E/K/D^14^). Many methods attempting to address the challenge of VUS determination have been developed over the last few years. Those methods can be divided to experimental methods, pure computational algorithms or a combination of both, and can utilize genome sequencing, transcriptome sequencing or proteomic profiling^15^.

Experimental methods for variant classification have the advantage of being independent of prior knowledge and can be used to test responses to MTAs. In one example^16^, a quantitative proteomics analysis was used to probe the proteomics of Triple Negative Breast Cancer (TNBC) to identify cancer subtypes and biomarkers. The proteomic profile was integrated with exome sequencing data to determine how protein expression is affected by genomic aberrations to initiate tumorigenesis. Another method^10^ involves sequencing and analysis of gene expression data, comparing perturbations induced by mutated and wild-type (WT) gene variants to label pathogenic variants in lung adenocarcinoma. The authors showed that resistance to erlotinib treatment which is caused by rare variants is MEK dependent. A recent method^17^, developed by Ng and colleagues, used two cell models that are growth factor dependent in addition to functional signaling profiles to probe the effect of more than 1000 genomic aberrations. Remarkably, the authors showed that their method is able to identify weak cancer drivers such as BRAF G466A and PI3KCA M1043I.

Pure computational approaches to classify VUS and identify cancer driving mutations mostly leverage evolutionary, functional and structural data, as well as data from clinical, family history, co-occurrence and other sources^18,19^. One of the first algorithms to be developed is PANTHER^20^. It works by calculating scores derived from position specific evolutionary conservation which is based on multiple sequence alignment of homologous proteins, to predict oncogenesis. While evolutionary conservation may be an informative prior, it may misclassify passenger mutations as drivers since the model does not have any disease context. FATHMM^21^ (Functional Analysis through Hidden Markov Models) aims to solve this issue and indeed achieved higher accuracy and precision on the same data set by incorporating a dataset of mutations found in inherited diseases. However, this biased the results towards mutations that are seen in observable inherited diseases, and the number of such diseases is considerably smaller than the number of somatic mutations. Another notable approach involves a Bayesian framework constructed from a set of publicly available in-silico predictors^22^ that reports an AUC (area under the ROC curve) of 0.997 when their ensemble was evaluated on data of 1161 missense mutations. NIPS (Network Integrated Predictor of deleterious protein Single amino acid polymorphism) is a structure-based approach that identifies deleterious mutations in tumor samples. It works by integrating data from several sources, including 3D protein-protein interface interactions, evolutionary conservation and network topology. While the study reports AUC of 0.93, it is still limited to cases where protein structures are available. One of the most comprehensive studies^8^ to characterize cancer driver mutations used an ensemble of 26 different algorithms and identified 299 driver genes and >3400 missense driver mutations; 60–85% of mutations were validated experimentally as probable cancer drivers.

DCNNs are a class of machine learning models that have gained a considerable amount of attention recently because of their superior performance in various machine learning tasks^23–25^. Such models have been successful at various classification, labelling and segmentation tasks in biomedical research. In cancer genetics, deepDriver^26^ is a notable example of the application of DCNNs for the task of cancer driver genes prediction. This study used a DCNN that was trained on tensors constructed from a combination of mutation-based features (such as the fraction of silent or missense mutations in tumor samples) and gene similarity network. Although the performance of the algorithm is high (AUC of 0.984 and 0.976 on breast cancer and colorectal cancer), predicting the role of individual mutations and VUS in particular, is beyond its scope. Another tool, Mut2Vec^27^, is an unsupervised approach for cancer driver prediction which is based on the popular Word2Vec^28^ class of models. In Mut2Vec, the model is trained on a set of cancer profiles to generate an embedding for each mutation, showing that passenger and driver mutations can be distinguished when the embeddings are clustered. Pathology is another field of cancer research that has gone through significant transformations by the recent advances in deep learning^29^. In one study^30^, a DCNN that was trained on slide images of sentinel lymph node biopsies was able to classify and label tumors with exceptional accuracy. Another study^31^ developed a DCNN trained on a dataset that integrated histology images and genomic biomarkers to predict time-to-progression outcomes. The authors showed that by integrating genomic data the median concordance index was significantly improved from 0.754 to 0.801. Another field that has successfully utilized deep learning is fluorescent microscopy. Wang *et al*.^32^ developed a Generative Adversarial Network for increasing the resolution of diffraction limited fluorescent microscopy images, wide-field images taken with low numerical aperture objectives, and confocal microscopy images which were able to achieve the resolution acquired with a stimulated emission depletion (STED) microscope. Christiansen *et al*.^33^ developed a neural network architecture that is composed of several sub-networks, each accepts as input a scaled version of the image. This network was shown to accurately predict the fluorescent labels of unlabeled microscopy images. The inputs to the network are patches of images taken with differential interference contrast, bright-field or phase contrast microscopy at multiple resolutions; the network outputs a vector of 256 intensity values for each of the pixels of the output image. This study demonstrated the effective use of a multi-scale network to create an artificial fluorescent labeling system that requires minimal experimental preparations and has much less impact on imaged cells.

**Figure 1:**
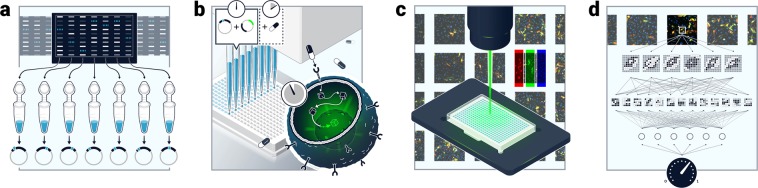
Pathway activation level prediction pipeline. (**a**) Mutations are collected from different sources, synthesized and verified using Sanger Sequencing, (**b)** HeLa cells are seeded in a 384-well Poly-L-lysine coated, transparent bottom plate and are transfected with plasmids carrying the desired mutation and an EGFP tagged reporter. Cells are incubated for 24 hours and then fixated using PFA. Alternatively, cells can be incubated with different inhibitors. (**c)** Cells are imaged using a fluorescent microscope, (**d)**. Images are inputted to a DCNN for analysis/training. With thanks to Elvire Thouvenot-Nitzan for the graphics design.

To the best of our knowledge, this is the first study in which DCNNs are applied, together with a novel dataset of more than 60,000 fluorescent microscopy images to determine the role of VUS in oncogenesis. During training, the network constructs a latent representation of pathway hyperactivation and uses it to quantify the level of oncogenicity of other mutations, which the network has never seen before. Similar solutions were implemented for problems such as predicting personal traits or estimating chronological age both from facial images^34,35^, where latent representations for people’s chronological age or for traits such as Intro/Extroversion are constructed during training and are used for trait quantification during inference. We trained our DCNN on a large set of fluorescent microscopy images of live cells transfected with a plasmid containing a fluorescently tagged mutant or WT gene and a fluorescently tagged downstream reporter that translocates into the nucleus upon pathway activation (Fig. 1). We show that our system accurately measures several known mutations as well as VUS activity levels. We further show that although the network has not been trained on images of MTA treated cells, it is able to predict responses of mutations to MTAs. Altogether these results establish a system that can be used for variant annotation and sensitivity to MTAs.

## Materials and Methods

### System overview

First (Fig. 1a), mutations are collected from different sources and are synthesized using the Q5 site directed mutagenesis kit (New England Biolabs, Cat #E0554S) and verified using Sanger sequencing. Next (Fig. 1b), HeLa cells are seeded in a 384-well Poly-L-lysine coated, transparent bottom plate. Twenty-four hours after seeding, cells are transfected with plasmids carrying the desired mutation and an EGFP tagged reporter. For the MAPK/ERK pathway the ERK2 reporter was used^36^, for the JAK-STAT pathways the STAT3 reporter was used^37^ in four repeats using the Fugene HD reagent (Promega, Cat. #E2312). After transfection, cells are incubated for 24-hours to allow adequate expression of the gene constructs. The plates were then fixated using 3% Paraformaldehyde, and a nuclear stain (DAPI) was performed. In the third step, (Fig. 1c) images of the plates are taken using a NIKON Ti eclipse microscope and NIS-elements software. Finally, in the last step, (Fig. 1d) images of wells seeded with cells transfected with selected known oncogenic mutations and wildtype forms of the same genes are inputted for a DCNN for training or inference.

#### Cell culture

HeLa cell line was obtained from ATCC (Rockville, MD) and were grown under standard condition for 14 passages at most. We used EMEM media supplied with 10% sera a (Gibco, LIFE) L-Glutamine, Sodium Pyruvate and antibiotics. FUGENE (promega) was used for transfection procedure according to manufacturer protocol. For the transfections we used Janus (PE) liquid handler system in 384 well plates, Poly-L-Lysin coated, with non-supplemented media. The raw images were obtained using automated NIKON Ti-Eclipse microscope coupled with an Andor Zyla 4.2 PLUS sCMOS camera and a LED-based SOLA light source.

### The dataset

Our dataset is composed of 65,698 multi-channel images of cells from individual wells from 384 well-plates that were transfected with plasmids carrying either mutated or WT KRAS, NRAS, HRAS, BRAF, MEK, cKIT or PDGFRa genes that were transiently expressed. The image data set contains 308 different gene variants (213 images per mutation on average) that were assigned one of 3 levels of certainty (activating, predicted to activate, VUS) regarding their oncogenicity according to the JAX-CKB^38^ database mutation classification system (See Supplementary Table S2 for a list of the tested mutations and their corresponding JAX-CKB classification). Each image in the dataset is composed of 3 color channels – red (610 nm), green (509 nm) and blue (461 nm). The green color corresponds to a GFP tagged reporter. For the KRAS, NRAS, HRAS, BRAF and MEK genes, the reporter was ERK2 and for cKIT and PDGFRa the reporter was STAT3. The red channel corresponds to mCherry which was used to tag the gene itself. The blue color channel corresponds to a DAPI stain that binds the DNA molecules in the nucleus. Overall, there were 3,543 ± 767 visible DAPI stained cells in the field of view (FOV) of each well on average, out of those 429 ± 159 (≈12%) were positive for mCherry (expressing the tested gene) indicating a successful transfection.

### DCNN Architecture design and implementation

**Figure 2:**
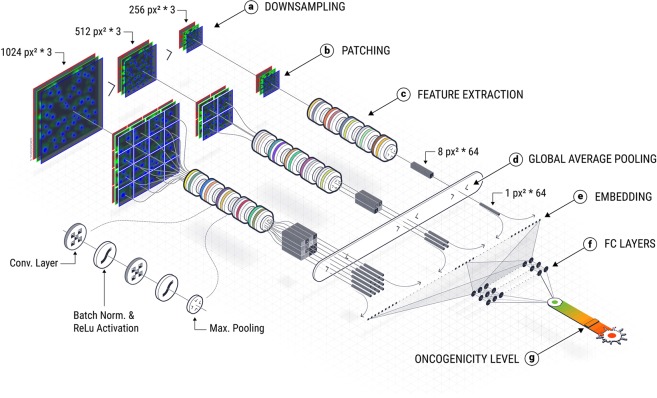
Deep Multi-Scale Network Architecture. (**a**) Down sampling – 3 versions of the image are created at 3 resolutions (1024 × 1024, 512 × 512, 256 × 256), and are fed into parallel computation paths. (**b)** Patching – each version of the image is broken down to 256 × 256 patches (the 256 × 256 remains as one patch). (**c)** Feature Extraction – The basic module that is used for feature extraction contains: 3 × 3 2D convolution with ReLU activation function, Batch Normalization and Max pooling with a 2 × 2 window. The feature extraction step has 5 such modules that are applied in succession to each patch. At the end of the feature extraction stage, each patch (256 × 256 image) is transformed to 8 × 8 × 64 matrix. (**d)** Global Average Pooling – For each 8 × 8 × 64 matrix the average across spatial dimension is calculated, outputting a vector V, s.t. $|V|=64$. (**e)** Embedding – the output of the global average pooling for all patches is flattened and concatenated. (**f**) Fully Connected Layers – the embedding is inputted to 3 fully connected layers (100 neurons each) with 20% dropout between each layer and ReLU activation function. (**g)** Oncogenicity Level – the final fully connected layer is connected to one output neuron with sigmoid activation. With thanks to Elvire Thouvenot-Nitzan for the graphics design.

We constructed a DCNN that follows a novel multi-scale architecture (Fig. 2). This class of models was demonstrated in several studies to have superior performance in image segmentation, labeling^33^ and classification^39^ (compared to other classes of models such as pixel-wise CNN or combined SVM and RF classifier).

The DCNN was trained using Tensorflow and Keras; data preparation and analysis was done in python, matplotlib and seaborn.

The computation path is composed of 7 main steps. First, to enable the network to operate on different scales, the input images are scaled to 3 different resolutions (Fig. 2a). Then, each image is broken down into patches of 256 × 256 (Fig. 2b), which reduces complexity and regularizes the network. During training, only features that are consistently present in many patches are selected. Subsequently, for each patch, features of increasing complexity are computed. This is done by 5 rounds, each composed of 2D convolutions with a 3 × 3 kernel, and an increasing number of convolution filters at each round (4,8,16,32,64), batch normalization^40^ and a 2 × 2 maximum pooling, outputting a 8 × 8 × 64 feature matrix (Fig. 2c). Next, we reduce the dimension of the feature matrix by applying global average pooling, an operation that averages features across the spatial domain (Fig. 2d), outputting a vector |*v*| = 64 per patch. Finally, all vectors representing all patches at all scales for one image are concatenated (Fig. 2e) and are inputted to a fully connected layer to cross correlate features across all patches and scales (Fig. 2f). The last fully connected layer is connected to an output neuron with a sigmoid activation function (Fig. 2g) that outputs values in (0,1), where 0 corresponds to images containing cells transfected with WT genes, and 1 corresponds to images containing cells transfected with a pathway activating oncogenic mutant.

## Results

### Training phase

**Figure 3:**
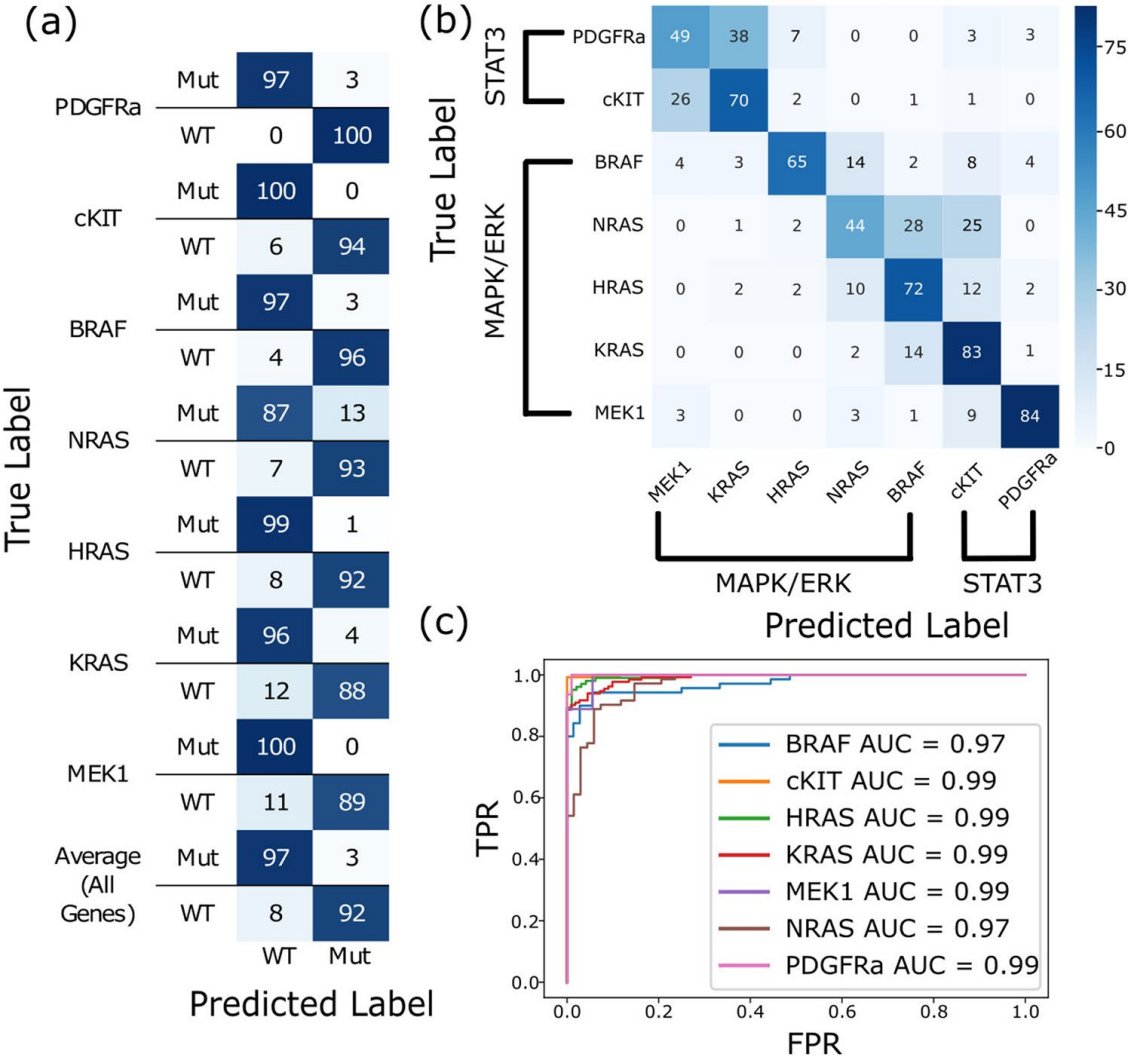
DCNN Performance Analysis shows high accuracy. (**a**) Confusion matrix for the WT and mutants of each of the genes. (**b**) Confusion matrix for mutant genes – test set (as % of total). Most cases of confusion occur on genes belonging to same pathway, in particular, RAS genes are most commonly confused. (**c**) ROC curve for the test set combining 7 different genes. (480 images of WT and a known oncogenic mutation for each).

Out of the data set of 7 genes, for which we have a total of 301 mutated variants and wildtype forms of each (see Materials and Methods for a description of the data set) we selected 8 mutated variants to be used as positive examples of pathway activation (one for each gene, except for cKIT for which we used 2 different mutations) and the wildtype form as negative examples for pathway activation. This subset was partitioned to training (60% of the images), validation (20% of the images), and test sets (20% of the images) with an additional stratification by well plate (all images from a plate belonged to the same set), to be able to assess the model generalization capabilities across experiments. (Summary of the data used for training, validation and test is in Table S1). An extensive hyperparameter tuning was performed and converged on the following hyperparameters - batch size of 32 images, Adam optimization method^41^ with a learning rate of 10^−4^. Following the training phase, we assessed the sensitivity (True Positive Rate) and specificity (True Negative Rate) of the network on the test set and found that it has high sensitivity and specificity across all genes and pathway reporters, with mean AUC of 0.98 (Fig. 3c) and average of 95% accuracy (Fig. 3a).

Next, we tested whether there was a difference in the pathway activation patterns induced by each gene by training the DCNN to predict with which gene the cells in the image were transfected. For that purpose, we added to the DCNN from the previous step an additional output layer with 7 neurons (the number of different genes in the data set), with a softmax activation function - $Softmax(x)=\frac{\exp ({z}_{i})}{{\sum }_{j}\exp ({z}_{j})}$ which computes a probability distribution over multiple output neurons, and categorical cross entropy as a loss function: $-\frac{1}{N}\sum _{i\in I}\sum _{g\in G}\,\log ({P}_{model}({y}_{i}\in {G}_{g}))$ (where *G* corresponds to the set of genes, and *I* the image dataset). Finally, we created an additional ground truth vector with one-hot encoding such that: *y*^*g*^_*i*_ = 1 _*g*_ and the images were not changed. Following the training phase, we assessed the accuracy and specificity of the network on a hold-out set. Trained to identify the unique phenological properties induced by each gene, the network achieved a mean of 66% accuracy (Fig. 3c) where most cases of confusion occurred between the 3 RAS homologs, and to some extent BRAF. Similarly, cKIT and PDGFRA were also commonly confused. We hypothesize that this is because they were assayed with a different reporter gene (GFP-STAT3) than the rest of the genes in the study (N/H/K-RAS, MEK, BRAF were all assayed with GFP-ERK2 as a reporter), and that the reporter genes themselves contain intrinsic properties that differ between each other.

### VUS Determination

We used the trained DCNN to annotate mutations that have not been functionally profiled (VUS), as well as known oncogenic mutations, all of which were not encountered by the network during training, test or validation. For that purpose, we used a data set of 301 mutated variants that were collected from the cBioPortal^42^ database. Each gene variant was given one of 3 labels that corresponds to the level of evidence regarding their involvement in tumorigenesis, according to the JAX Clinical Knowledgebase (JAX-CKB)^38^: **activating**- peer-reviewed published literature demonstrating functional evidence that the gene alteration present results in increased intrinsic activity of the protein; **predicted to be oncogenic**- the specific type of gene alteration as well as its location is similar to other alterations in the same gene that have been functionally characterized as a gain of function within peer-reviewed published literature; and **unknown**- there is no peer-reviewed published literature demonstrating the gene alteration present affects the intrinsic activity of the protein.

We synthesized plasmids carrying each of the gene variants from the data set and the same reporter that was used for the gene during training, transfected and imaged them as was described above. The resulting images were inputted to the trained DCNN and the level of predicted pathway activation was determined. Table 1 summarizes the predictions for each label and each gene that was tested. A mutation was determined to be active if its mean prediction value, calculated over all fluorescent microscopy images was above the sigmoid middle point of 0.5. Out of the 301 tested mutations in all 7 genes, JAX-CKB classified 81 as activating, 24 as predicted to activate, and 196 as VUS. The “activating” class is the only class that can be used to validate the accuracy of our platform, since it contains only experimentally validated mutations. Remarkably, our system was able to correctly predict the pathway activation status of 75/81 (92.6%) of those experimentally validated, activating mutations (Table 1). Additionally20/24 (83.3%) of the variants labeled as “predicted to activate” (Table S2) And 63/196 (32.1%, Table 1) of the mutations that were labeled as VUS are predicted by our system to be pathway activating, hinting to their potential oncogenicity.

**Table 1:** Summary of CNN output.

Classification	Gene	% predicted to be active (N/total)
Activating mutations	BRAF	95% (19/20)
	KRAS	95.2% (20/21)
	HRAS	88.9% (8/9)
	NRAS	88.9% (8/9)
	MEK1	100% (2/2)
	cKIT	84.2% (16/18)
	PDGFRa	100% (2/2)
	Total	92.6% (75/81)
VUS	BRAF	28.3% (15/53)
	KRAS	60% (15/25)
	HRAS	9.5% (2/21)
	NRAS	20% (2/10)
	MEK1	No VUS in dataset
	cKIT	33.7% (28/83)
	PDGFRa	25% (1/4)
	Total	32.1% (63/196)

Summary of DCNN prediction values for mutations determined by JAX-CKB to be pathway activating and mutations that were labeled as VUS. Fluorescent microscopy images of HeLa cells transfected with a mutated gene variant were inputted to the trained DCNN; prediction values were averaged for each mutation. 0.5 was used as a cutoff for determining whether a mutation is pathway activating or benign.

**Figure 4:**
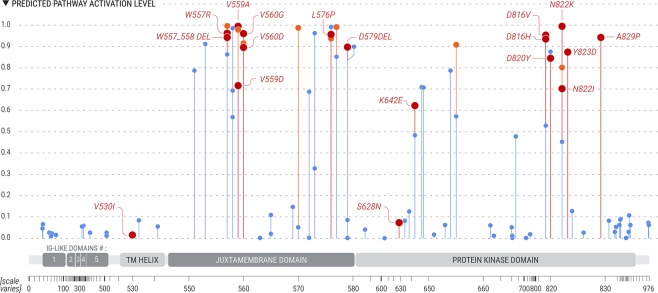
cKIT mutations activity profiles do not show dependence on spatial localization. Lollipop plot of the mean DCNN output for 110 different cKIT mutations. Colors were assigned to lollipops according to the JAX-CKB annotation for each of the mutations: Red for mutations annotated as oncogenic, orange for mutations annotated as predicted oncogenic and blue for VUS. For simplicity, only verified oncogenic mutations are labeled. Hight of the bar denotes its measured activity. X-axis relates to the amino acid position of the gene (not to scale). With thanks to Elvire Thouvenot-Nitzan for the graphics design.

As an example, the output of the network for each of the surveyed variants of cKIT, 110 in total, is presented in Fig. 4. As can be seen, most cKIT mutations tested are concentrated in the juxtamembrane and protein kinase domains, resembling the relative distribution of mutations in different cancer types. Several cKIT VUS were predicted by our system to lead to pathway activation and could be novel cancer drivers. For example, cKIT Y553S and P551L are both predicted by our system to be active and lie within the juxtamembrane domain. P551L has been identified in sequencing studies^43^ but has not been biochemically characterized, while Y553S has not been functionally analyzed but has been associated with imatinib resistance^44^. Similarly, cKIT V654A which lies in the kinase domain has conflicting evidence regarding its pathway activation capabilities. It was found to lead to increased proliferation of cultured cells but not to factor independence and has been described as a secondary drug resistance mutation^45^.

In the case of KRAS, 51 variants were tested most of which are concentrated in the phosphate binding loop, base binding loops and switches I,II, with G13,G12 and Q61 being the positions with the highest incidence of activating mutations (Supplementary Fig. S1). The high incidence of active VUS in KRAS (60%) compared to HRAS (9.5%) and NRAS (20%) stems from the large number of G12-G13 deletion/insertion variants that were tested only for KRAS and were predicted to be active. The VUS tested range from small deletions such as KRAS V152del, to missense mutations such as L23R, N116H and indels such as G12_G13_Del_Ins_DC, all with little to no evidence regarding their oncogenic activity. Interestingly, the KRAS mutation N116H has been known to increase the nucleotide exchange rate of KRAS^46^ and therefore activate MAPK signaling. Similarly, KRAS L23R was predicted by our system to be pathway activating. Although it was identified in several sequencing studies of cancer patients^47,48^, it does not lie within any known functional domains of KRAS and has not been biochemically characterized. KRAS V152del is another rare mutation which was predicted by our DCNN to be pathway activating, and although a different mutation - V152G was identified in a recent sequencing study^49^ as active, the V152del variant lacks any evidence regarding its activity.

Of the six genes analyzed, BRAF had lowest concordance between literature and our results (20% of mutations predicted to be active correctly analyzed, Supplementary Table S2). We therefore analyzed these false negatives and found that the only false negative in the known activating mutation class (G466V) and 3 of the 4 false negative in the predicted to activate class (BRAF D594N, G466R, G596R) were classified recently as a distinct class of BRAF mutations (BRAF class III) that differ significantly from the V600E\K\D mutations and were found to possess basal kinase activity that is lower when compared to WT BRAF, or lack kinase activity entirely^50,51^. Moreover, biochemical studies predict that this class of mutants would require upstream activation of MAPK for pathway activity and tumorigenesis^52^. Therefore, this class of mutations may have been missed since our system was trained to identify only mutations that directly lead to pathway activation and do not depend on other mutations to induce tumorigenesis. BRAF V600M is the 4^th^ false negative class of “predicted to be activating”. It lies within the activation segment of the kinase domain of BRAF, at the same position of other highly activating mutations such as BRAF V600E\K. However it was shown to cause only intermediate increase in kinase activity in cell culture^52^. The mean prediction value determined by our network for V600M (0.47) is in concordance with the intermediate kinase activity reported by the literature, providing an additional evidence for the accuracy of our platform.

Three additional false negatives that are known pathway activators are RAS mutations: KRAS T58I, NRAS G60E, HRAS G13S – all lie in the GTP binding domain of each of the RAS proteins and are characterized as MAPK pathway activating and proliferating inducing mutations^53–55^. Although these mutations are below the cutoff determined for pathway activation (0.5), all have a mean pathway activation score significantly higher than their wildtype variants (0.33, 0.38, 0.18 respectively, student’s t-test p-value < 0.002 for all variants compared to wildtype mean activation scores across all well images). The last 2 false negatives are cKIT variants S628N and V530I. Both were documented as pathway activating; S628N lies within the protein kinase domain (exon 13) of the protein and results in constitutive Kit phosphorylation and activation of downstream signaling, and is transforming in cell culture^56^. V530I lies within the transmembrane domain of the Kit protein and confers a gain of function on the protein, as indicated by constitutive phosphorylation of cKIT and activation of signaling in cell culture^57^.

Concluding, our method shows remarkable ability in identifying pathway activating mutations, with a success rate of 92.6% over the class of known pathway activating mutations and 83.3% over mutations which are predicted to be activating based on similar or proximal alterations. The success rates increase to 93.8% and 95.4% respectively when class III BRAF mutations are excluded. Finally, almost a third (32.3%) of the alterations that were labeled as unknown were found to be active, a finding that demonstrates the importance of functionally testing all identified mutations.

### Prediction of drug responses

**Figure 5:**
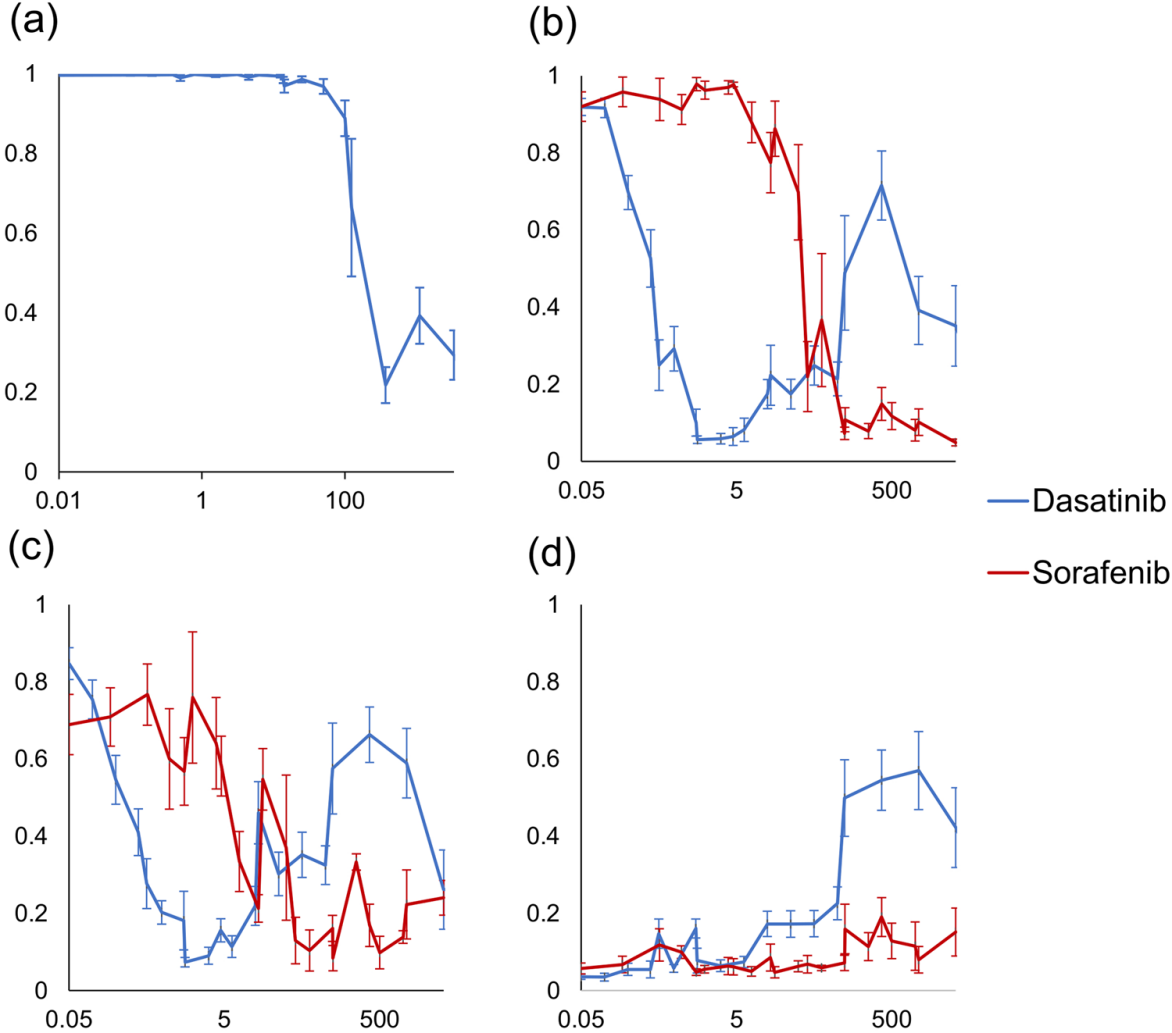
cKIT Drug Response Curves show differential sensitivity to different inhibitors. Cells transfected with a D816V (**a**), W557_558 del (**b**) W557R (**c**) or WT (**d**) cKIT were incubated with increasing concentrations of dasatinib or sorafenib for 18 hours, imaged and served as an input to the trained classifier. The network output was recorded and plotted as a function of the drug concentration. Each point in the plot corresponds to the mean output value of the DCNN for images of wells with the same concentration, error bars corresponds to the standard error of the mean.

One of the main features of our platform is the ability to test drug responses on different gene variants and pathways. To test the accuracy of this capability, we tested the response of 3 different cKIT alterations (W557R, W557_558 Del, D816V) and cKIT WT to sorafenib or dasatinib, FDA approved drugs and potent cKIT inhibitors^58,59^. All the cKIT alterations are annotated by JAX-CKB as activating and identified as activating in our system (Fig. 4). Each of the 3 cKIT gene variants as well as the WT form were expressed, and the cells were incubated for 18 hours with either sorafenib or dasatinib in increasing doses. We inputted the images to the trained DCNN and for each drug concentration recorded the mean network output across all images for each mutation in each concentration (Fig. 5). As can be seen, our system clearly identified a dose dependent decrease in pathway activation level for each of the cKIT alterations and for both Dasatinib and Sorafenib, with Dasatinib showing significant drop in predicted pathway activation levels in lower concentrations than Sorafenib, which is consistent with previously published literature^60^.

One mutation, D816V, (Fig. 5a) was predicted by our network to be more resistant to dasatinib than the rest of the mutations, as it shows a decrease in activity only at higher concentrations (100nM–1uM). This is consistent with previous studies showing decreased sensitivity of D816V to dasatinib^61^. For cKIT W557_558Del (Fig. 5b) and W557R (Fig. 5c) the mean network output reaches values lower than 0.2 at 1–10 nM for dasatinib and 100 nM for Sorafenib, while the output for the cKIT WT (Fig. 5d) remains close to 0 for both drugs at these concentrations. Interestingly, we also observed an increase in predicted pathway activity level at concentrations higher than 10 nM in the dasatinib treated cells, which was not apparent for sorafenib and only apparent to a lesser degree for D816V. We hypothesize that this outcome results from off-target effects at high concentrations of the drug. Such effects have been documented previously for dasatinib^62,63^.

## Discussion

We present here a novel method for determining the functional role of VUS and their response to targeted therapies, that can be used as a tool to guide the development of targeted therapies. Our method synergizes an experimental functional assay with a computational framework composed of a DCNN, that was trained on several thousands of fluorescent microscopy images of cells transfected with mutated or WT genes (BRAF, cKIT, HRAS, KRAS, NRAS, PDGFRa) to identify the activity of mutations annotated as VUS. The method involves fluorescent tagging of 3 key components: the mutated protein itself, a downstream signaling protein fused to GFP (ERK2 or STAT3) and nucleic DNA (DAPI staining). The novel network architecture we presented here has been carefully selected after considering many existing state of the art alternative architectures in the field of image recognition, such as ResNet^64^ and Inception^65^. Those networks are composed of millions of free parameters and are usually trained on large and diverse image datasets^66^ containing more than a million images. Using this class of network architectures on a smaller dataset such as ours, most commonly leads to overfitting. There are several advantages to the architecture presented in this study: First, the number of free parameters is considerably smaller, a few hundred-thousands of parameters (compared to millions in the above-mentioned architectures). Second, similarly to ResNet, our architecture enables the cross-correlation of low-level and high-level features, learned from different resolutions of the same image. Third, our network avoids the vanishing gradient phenomenon that frequently occurs in deep neural networks by adopting an architecture that is composed of several shallow networks. A similar interpretation has been described by Veit and colleagues for residual neural networks^67^.

Most state-of-the-art architectures mentioned above are frequently used to extract features from images of other domains (such as skin lesions^68^) for subsequent learning tasks in an approach called transfer learning^69^. The performance of a classifier resulted from transfer learning is directly related to the similarity between the domain of the source dataset, on which the network was originally trained, and the target dataset. In our case however, the degree of similarity between the source dataset (e.g. ImageNet) and the target dataset (fluorescently labeled HeLa cells) is considerably low and indeed, transfer learning approaches resulted in classifiers with degraded performance (data not shown).

We have shown that our system not only recognizes whether a gene variant leads to pathway activation but is also able to recognize in most cases the type of gene that is expressed in the cells and the type of reporter used in the assay. Features learned by deep neural networks are notoriously difficult to interpret, as they are often immensely complex tensors composed of many dimensions. Recent studies^70^ in the field of explainable deep neural networks should bridge this gap and may help explain the unique changes that each variant and reporter induces on cells.

A frequently observed phenotype in tumorigenesis is pathway hyper-activation which results in a range of phenotypes^71^. Compared to methods which are purely computational and can identify pathway activating mutations using various in-silico approaches, our method has the advantage of being able to predict a dose dependent pathway activation level change, which is currently out of scope for purely computational approaches.

Indeed, we show that our system is currently only able to identify cancer drivers and annotate VUS that lead to pathway hyperactivation. However, the challenge of VUS determination remains, as there are modes of operations that were beyond the scope of this study. For example, BRAF class III mutations such as BRAF D594G, G466V, G596R, G466E mutations which were predicted to be inactive by our system but are known cancer drivers. These alterations constitute an entirely different class of BRAF mutations that lead to pathway activation using a different mechanism than V600 mutations^52^ that served as our training set. This class of mutations work in tandem with other aberrations to generate a malignant phenotype. Specifically, they require a dysregulated RAS in order to hyperactivate the ERK pathway^14^. Determining the role of such mutations without the context of its co-occurring mutations may lead to false predictions. Another aspect that should be addressed by future studies, is tissue specificity of some oncogenic gene variants, some genetic aberrations function as cancer drivers only in specific tissues, such as the loss of function of BRCA which can be found only in breast and cervical cancers^72^.

The biological mechanism that was addressed in this study included only reporters whose main property is that they shuttle between the cytoplasm and nucleus. Both ERK2 and STAT3 are translocated into the nucleus following their phosphorylation. However, there are many other biological mechanisms that have been correlated to oncogenic mutations, for example changes in expression levels or translocation of proteins between different compartments of the cell. It has long been known that HER2 overexpression, which occurs in 15–30% of breast cancers and 10–30% of gastric/gastroesophageal cancers is a cancer driving alteration^73^. Other than HER2, there is a substantial amount of evidence that overexpression of MYC, MYCN, ER and EGFR is also involved in disease^74^. Changes in subcellular localization have also been characterized as a cancer phenotype. In one example, MUC1, a membrane bound protein which is expressed at the apical borders of glandular epithelial cells, is overexpressed in the nucleus as well as the entire cell surface, cytoplasm and mitochondria. Translocation of MUC1 to the mitochondria leads to apoptosis suppression by attenuating caspase-3 activation as well as the release of cytochrome-c^75^. Dysregulation of cell death signals, some of which are mediated by the BCL-2 protein family^76^, may also serve as reporters in similar circumstances. For example^77^, BCL-2-related ovarian killer (BOK) was found to be significantly depleted in colorectal tumors, and its levels also accurately predicted clinical outcome.

We have also demonstrated the capability of our system to predict drug responses in a dose dependent manner. This ability, coupled with the annotation of VUS activity can be leveraged for several clinically relevant uses, for example, optimizing the MTA clinical development process and improving patient’s treatment recommendations. In the case of development of novel MTA’s, it has been shown that the patient mutational landscape varies significantly^78^. Moreover, current drug development processes usually consider only a handful of highly frequent mutations as a model system. However, it has been shown that the same inhibitor can have significantly different efficacies on different mutations in the same gene, with some prominent examples in BRAF^79^ and cKIT^80^. We therefore suggest that considering these large numbers of mutations and their differential vulnerabilities to inhibitors early in the MTA development process will allow a much higher rate of success. The second aspect in which this system can be utilized is optimizing treatments for cancer patients. It has recently been shown that more comprehensive interpretation of genetic profiles can both improve the matching of patient to available treatments^81^ as well as new drug combinations^82^. The annotation of the many VUS found in patient genomic profiles will increase the matching score and therefore improve patient outcomes.

In summary, we have presented in this study a system that can determine the level of pathway activation of a wide range of gene variants and predict the response of those to different MTA’s. Future work will need to focus on expanding the capabilities of this model, for example, by training on more genes, mutations and reporters, increasing the robustness of the network and using different types of reporters.

## Supplementary information

Supplementary Dataset 1.
