# Supplementary material for: A Novel System for Functional Determination of Variants of Uncertain Significance using Deep Convolutional Neural Networks
Source: Sci Rep. 2020 Mar 6;10:4192. doi: 10.1038/s41598-020-61173-1 (PMC7060242; doi:10.1038/s41598-020-61173-1)
Supplement: Supplementary file 1 — Supplementary Dataset 1. [file 41598_2020_61173_MOESM1_ESM.docx]

**A Novel System for Functional Determination of Variants of Uncertain Significance using Deep Convolutional Neural**

**Networks**

# Lior Zimmerman^1^, Ori Zelichov^1^, Arie Aizenmann^1^, Zohar Barbash^1^, Michael Vidne^1^, and Gabi Tarcic ^1*^

# Supplementary Material


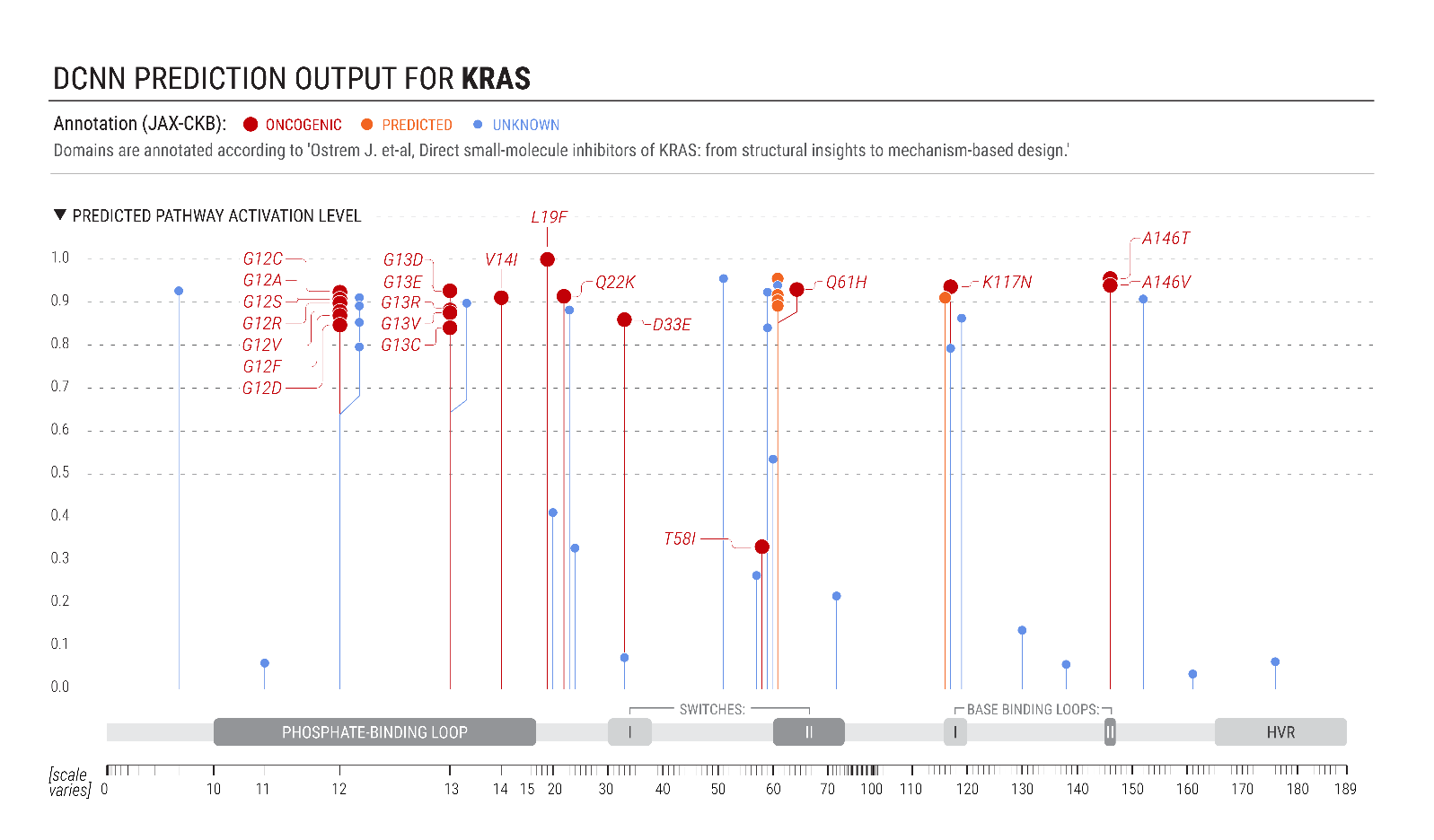


**Figure S1.** **KRAS VUS Determination**. Lollipop plot of DCNN output with respect to microscopy images of cells containing KRAS. Each lollipop represents the mean DCNN output of repeated experiments of the same mutation. Colors were assigned to lollipops according to the JAX-CKB annotation for each of the mutations: Red for mutations which were verified as oncogenic, orange for mutations which do not have direct evidence for their oncogenicity but are similar to known oncogenic variants, blue for VUS . For simplicity, only verified oncogenic mutations are labeled. Hight of the bar denotes its measured activity. X-axis relates to the amino acid position of the gene (not to scale).

# Table S1: The number of images per each mutation that was used for the training, test and validation

| Gene | Mutation | Number of images |
| --- | --- | --- |
| BRAF | V600E | 1,990 |
|  | WT | 1,704 |
| MEK1 | K57N | 669 |
|  | WT | 797 |
| KRAS | G13D | 2,317 |
|  | WT | 2,362 |
| HRAS | Q61R | 1,048 |
|  | WT | 944 |
| NRAS | Q61R | 1,520 |
|  | WT | 1,091 |
| PDGFRa | D842V | 246 |
|  | WT | 241 |
| cKIT | D816V | 1,435 |
|  | W557_558 Del | 1,396 |
|  | WT | 1,713 |
| **Total** |  | **19,474** |

# Table S2 – Summary of CNN output for mutations labeled as “predicted to be pathway activating” by JAX-CKB

| Gene | % predicted to be active (N/total) |
| --- | --- |
| BRAF | 20% (1/5) |
| KRAS | 100% (5/5) |
| HRAS | N/A |
| NRAS | 100% (6/6) |
| MEK1 | N/A |
| cKIT | 100% (8/8) |
| PDGFRa | N/A |
| Total | 32.1% (20/24) |
